# Long-Term Survival after Complete Resection of Local Duodenal Recurrence following Colon Cancer Surgery Using Laparoscopic and Endoscopic Cooperation: A Case Report

**DOI:** 10.70352/scrj.cr.26-0565

**Published:** 2026-10-24

**Authors:** Chizu Kameda, Yasunori Oda, Shunichi Takahata, Eishi Nagai, Yuji Nakafusa

**Affiliations:** 1 Department of Surgery, Japanese Red Cross Fukuoka Hospital, Fukuoka, Fukuoka, Japan; 2 Department of Surgery, Kyushu Railway Memorial Hospital, Kitakyushu, Fukuoka, Japan

**Keywords:** duodenal local recurrence, colorectal cancer, laparoscopic and endoscopic cooperative surgery, local resection, long-term survival

## Abstract

**INTRODUCTION:**

Laparoscopic and endoscopic cooperative surgery (LECS) enables accurate intraluminal localization and limited full-thickness resection while preserving gastrointestinal continuity. Although LECS has been used for selected primary duodenal tumors, its application to a postoperative duodenal recurrence of colorectal cancer has rarely been described. We report a case of localized duodenal recurrence after a right hemicolectomy that was completely resected using laparoscopic and endoscopic cooperation.

**CASE PRESENTATION:**

An 83-year-old man underwent a laparoscopic right hemicolectomy with a D3 lymph node dissection for advanced ascending colon cancer. Histopathological examination demonstrated a microscopically positive dissected margin on the duodenal side, indicating a microscopically positive margin (R1) resection. Nine months later, surveillance upper gastrointestinal endoscopy and contrast-enhanced CT revealed a solitary lesion in the second portion of the duodenum without distant metastasis or radiological pancreatic involvement. Endoscopic ultrasonography showed a lesion extending to the fourth layer. An upper gastrointestinal contrast study was not performed; instead, the location and local extent of the lesion were assessed by upper gastrointestinal endoscopy, endoscopic ultrasonography, and contrast-enhanced CT. Because the multimodal assessment indicated that complete local excision was technically achievable, LECS was performed. Intraoperative endoscopy delineated a wider intraluminal extent than was apparent from the serosal surface and guided an en bloc full-thickness resection. The defect was closed in the short-axis direction by a continuous full-thickness suturing. Intraoperative endoscopy confirmed the luminal patency and a negative leak test. Histopathological examination showed an adenocarcinoma involving the submucosa and muscularis propria without duodenal mucosal involvement. The adjacent muscularis propria was inverted toward the lumen, suggesting inward progression from the outer aspect of the duodenal wall. Together with the previous R1, these findings supported a localized recurrence from microscopic residual disease. The resection margins were negative, and the patient has remained disease-free for more than 5 years.

**CONCLUSIONS:**

Laparoscopic and endoscopic cooperation enabled precise definition of the recurrent lesion, complete full-thickness local resection, and secure closure while preserving duodenal continuity. This approach may be useful for strictly localized duodenal recurrence when careful preoperative assessment indicates that complete local excision is achievable.

## Abbreviations

EUS
: endoscopic ultrasonography
FDG-PET
: fluorodeoxyglucose PET
LECS
: laparoscopic and endoscopic cooperative surgery
R1
: microscopically positive margin

## INTRODUCTION

LECS was originally introduced by Hiki et al. for gastric gastrointestinal stromal tumors to permit precise local resection while preserving gastric function.^1)^ The combined approach provides endoscopic visualization of the intraluminal margin and laparoscopic control of full-thickness resection and closure, thereby limiting unnecessary tissue removal and postoperative deformity.^2,3)^

LECS has subsequently been applied to selected non-ampullary duodenal tumors. Previous studies have demonstrated its technical feasibility and acceptable safety when performed by teams experienced in both advanced endoscopy and laparoscopic surgery.^4–6)^ In the duodenum, accurate localization and secure closure are particularly important because the narrow lumen and exposure to bile and pancreatic juice increase the clinical consequences of stenosis or leakage.

Metachronous localized duodenal recurrence after colorectal cancer surgery is extremely rare, and the role of limited resection in this setting has not been established. We report a patient with an isolated duodenal recurrence arising after an R1 resection for ascending colon cancer. Laparoscopic and endoscopic cooperation enabled complete local excision, and the patient remained disease-free for more than 5 years.

## CASE PRESENTATION

An 83-year-old man presented with a 1-month history of progressively worsening abdominal pain and generalized fatigue. On admission, laboratory evaluation revealed severe anemia with a hemoglobin level of 7.4 g/dL. Total colonoscopy revealed an advanced tumor in the ascending colon, which was considered to be responsible for the severe anemia. Biopsy specimens obtained from the tumor revealed moderately differentiated adenocarcinoma of the colon. Preoperative contrast-enhanced CT showed no evidence of distant metastasis and suggested serosal invasion of the primary ascending colon cancer, whereas no definite radiological evidence of direct invasion into the duodenum was identified (**Fig. 1**).

**Fig. 1:**
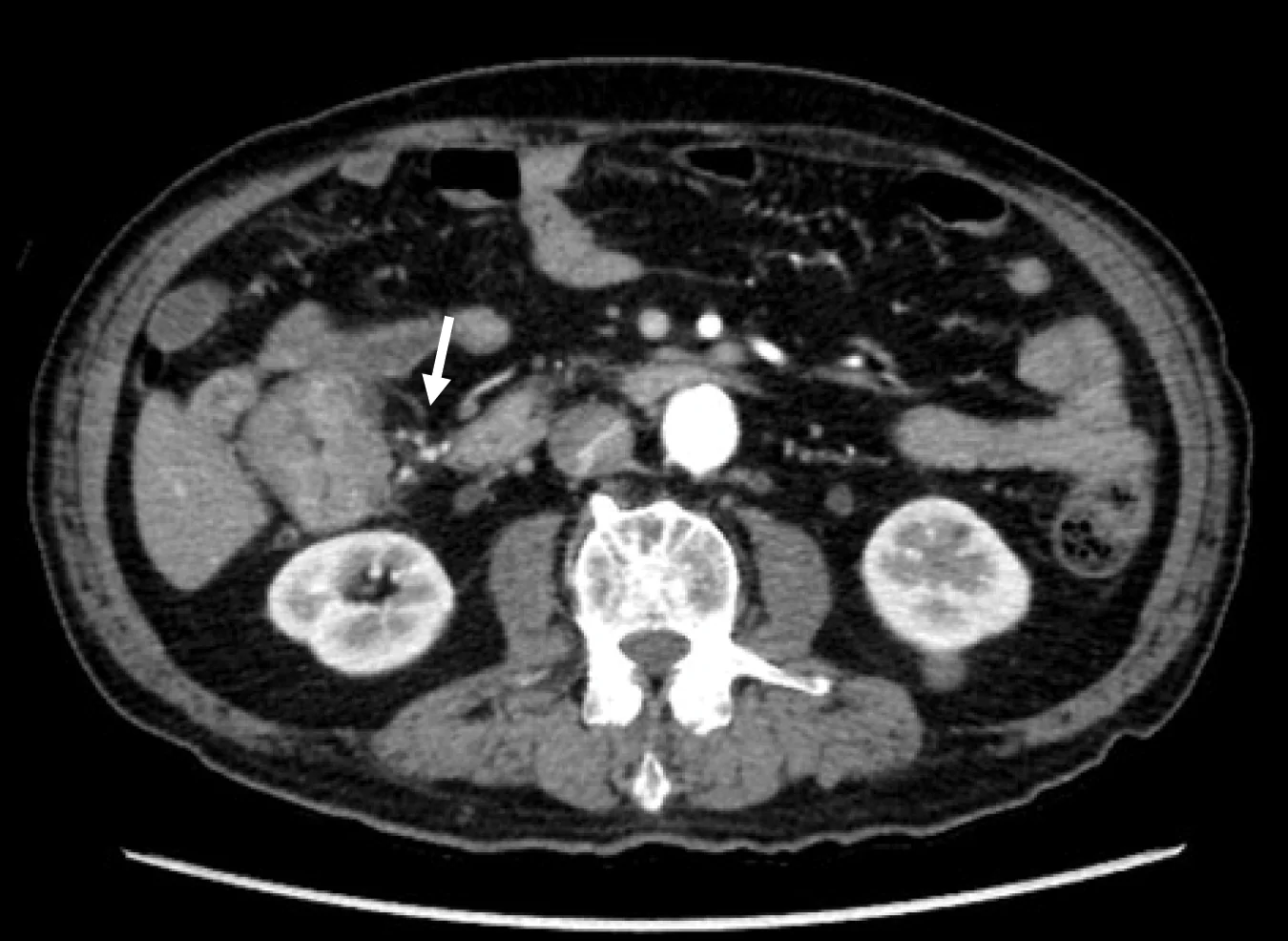
Preoperative contrast-enhanced CT before the initial surgery. Contrast-enhanced CT showed the primary ascending colon cancer with suspected serosal invasion. No definite radiological evidence of direct invasion into the adjacent second portion of the duodenum was identified (arrow).

The patient underwent a laparoscopic right hemicolectomy with D3 lymph node dissection. During the initial surgery, the primary ascending colon tumor was found to be firmly adherent to the second portion of the duodenum (**Fig. 2**). The duodenal wall was preserved without apparent injury after careful sharp dissection along the adherent interface. No operative finding specifically suggested that the duodenal involvement was caused by a metastatic lymph node.

**Fig. 2:**
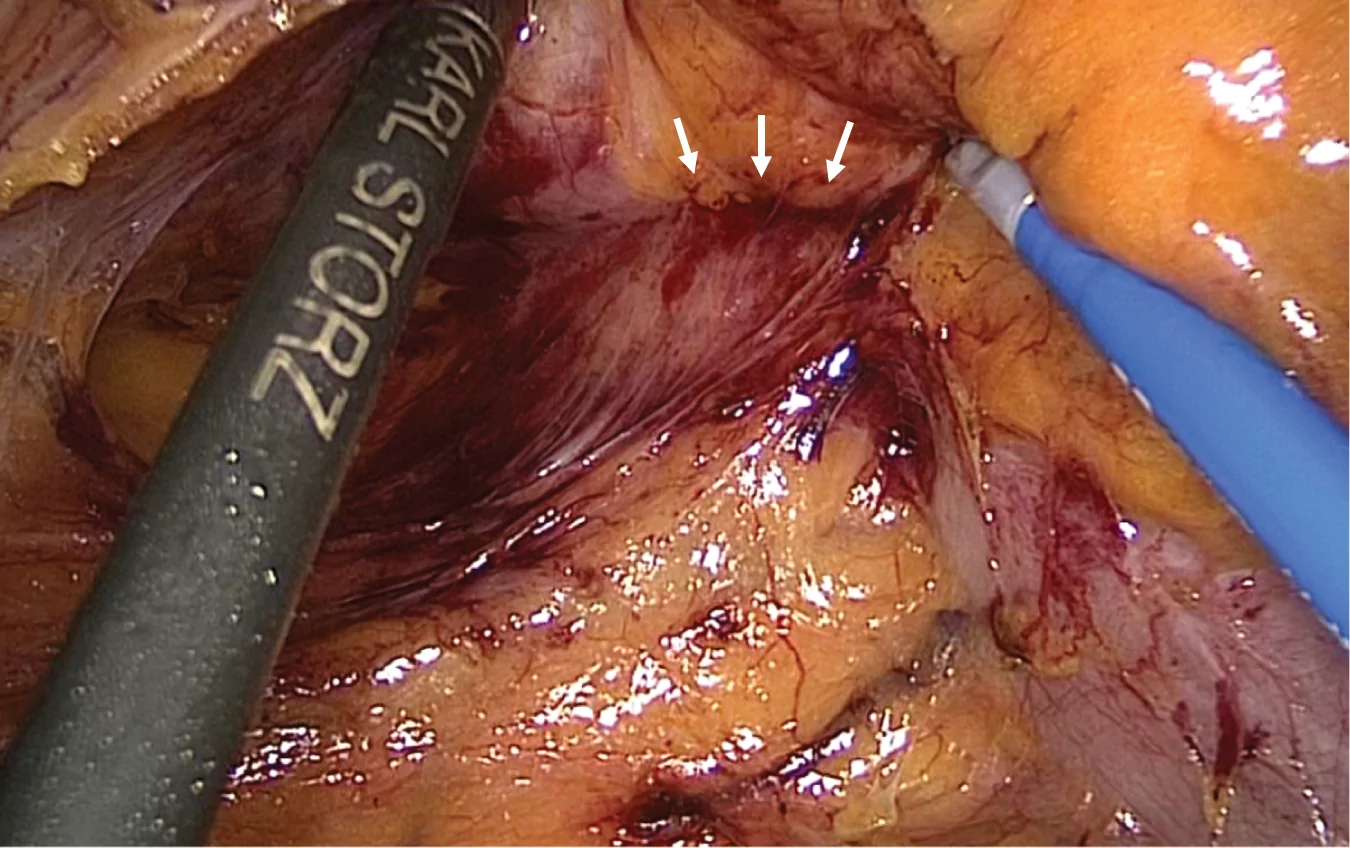
Intraoperative findings during the initial right hemicolectomy. The primary ascending colon tumor was firmly adherent to the second portion of the duodenum (arrows).

Histopathological examination revealed adenocarcinoma of the ascending colon, classified as pT4aN0M0 pathological Stage IIB (AJCC 8th edition). Microscopic evaluation revealed a microscopically positive dissected margin on the duodenal side, indicating R1 resection.

Given the high risk of local recurrence associated with microscopic residual disease, postoperative systemic chemotherapy with capecitabine was administered for 6 months.

Nine months after the initial colectomy, surveillance upper gastrointestinal endoscopy revealed a submucosal tumor with a central depression in the second portion of the duodenum (**Fig. 3**). Biopsy specimens from the depressed area revealed a moderately differentiated adenocarcinoma. EUS revealed a 20 × 16 mm heterogeneously hypoechoic lesion extending to the fourth layer (**Fig. 4**).

**Fig. 3:**
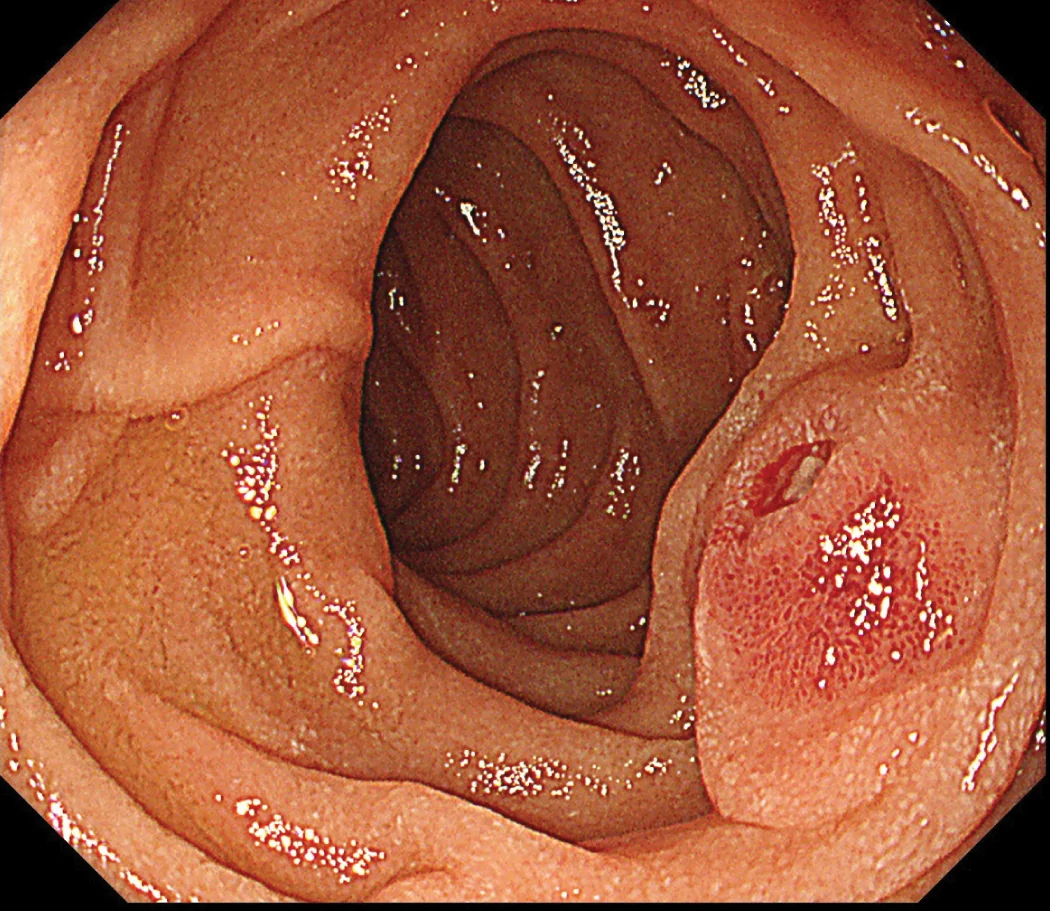
Esophagogastroduodenoscopy revealed a submucosal tumor-like lesion with a central depression in the second part of the duodenum. Biopsy histology revealed moderately differentiated adenocarcinoma.

**Fig. 4:**
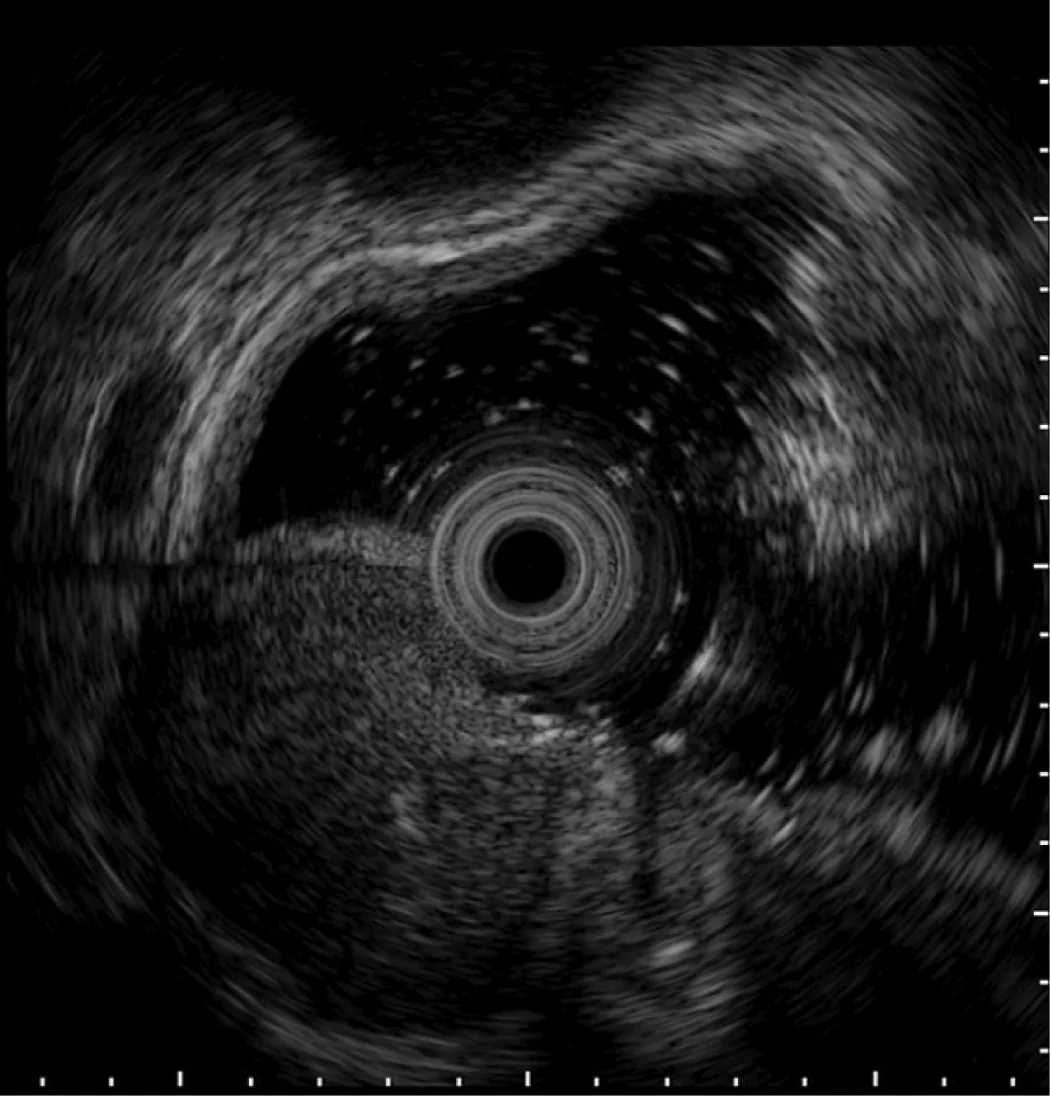
Endoscopic ultrasonography revealed a heterogeneous hypoechoic mass extending to the fourth layer.

FDG-PET revealed increased uptake confined to the duodenal lesion, with no abnormal accumulation in other organs (**Fig. 5**). Contrast-enhanced CT showed no distant metastasis, significant lymph node enlargement, or radiological evidence of pancreatic parenchymal invasion. An upper gastrointestinal contrast study was not performed; the location and local extent of the lesion were assessed by upper gastrointestinal endoscopy, EUS, and contrast-enhanced CT. The lesion was located in the second portion of the duodenum opposite the major papilla and was considered confined to the duodenal wall. Based on the previous R1 on the duodenal side, the endoscopic and imaging findings, and the absence of disseminated disease, metachronous localized duodenal recurrence was suspected. Because complete local excision appeared technically feasible, LECS was planned.

**Fig. 5:**
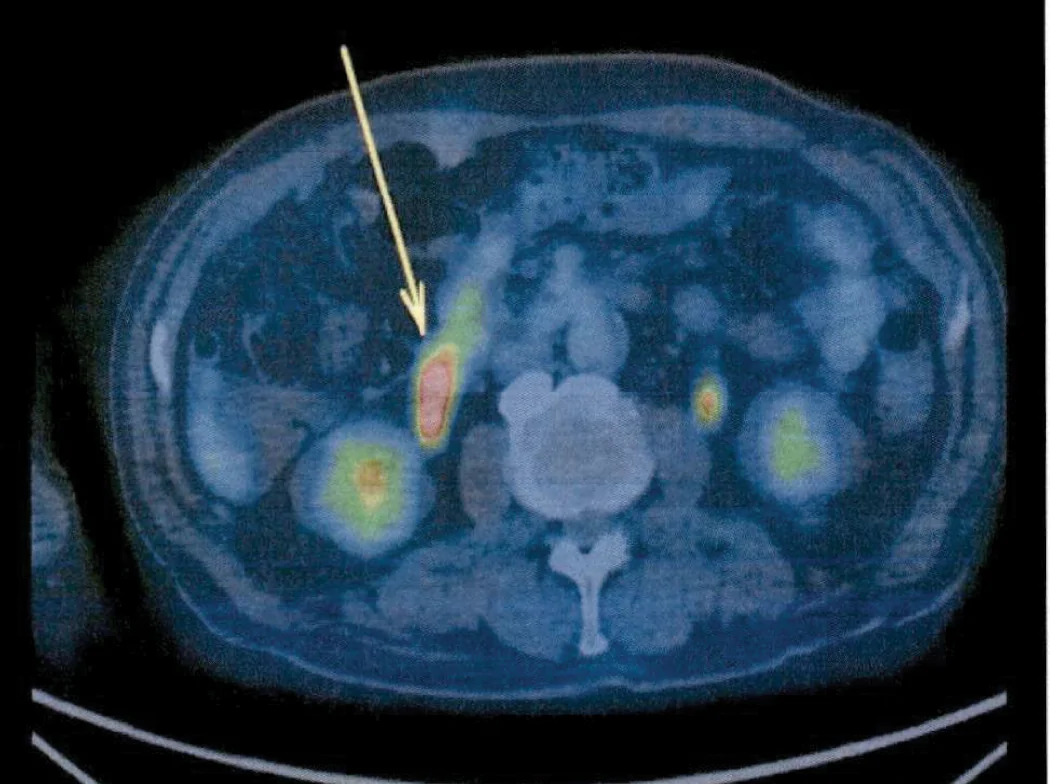
PET demonstrated the increased uptake of fluorodeoxyglucose in the second part of the duodenum (arrow).

Under general anesthesia, the patient was placed in the supine position. Five ports were inserted: a 12-mm umbilical camera port for pneumoperitoneum, a 12-mm port in the left upper abdomen, a 5-mm port in the right upper abdomen, and two 5-mm ports in the bilateral lower quadrants. Serosal retraction was observed in the second portion of the duodenum, corresponding to the suspected lesion (**Fig. 6A**). During duodenal mobilization, a portion of the adjacent retroperitoneal tissue was intentionally left attached to the duodenal wall and resected en bloc with the lesion to secure an adequate surgical margin (**Fig. 6B**).

**Fig. 6:**
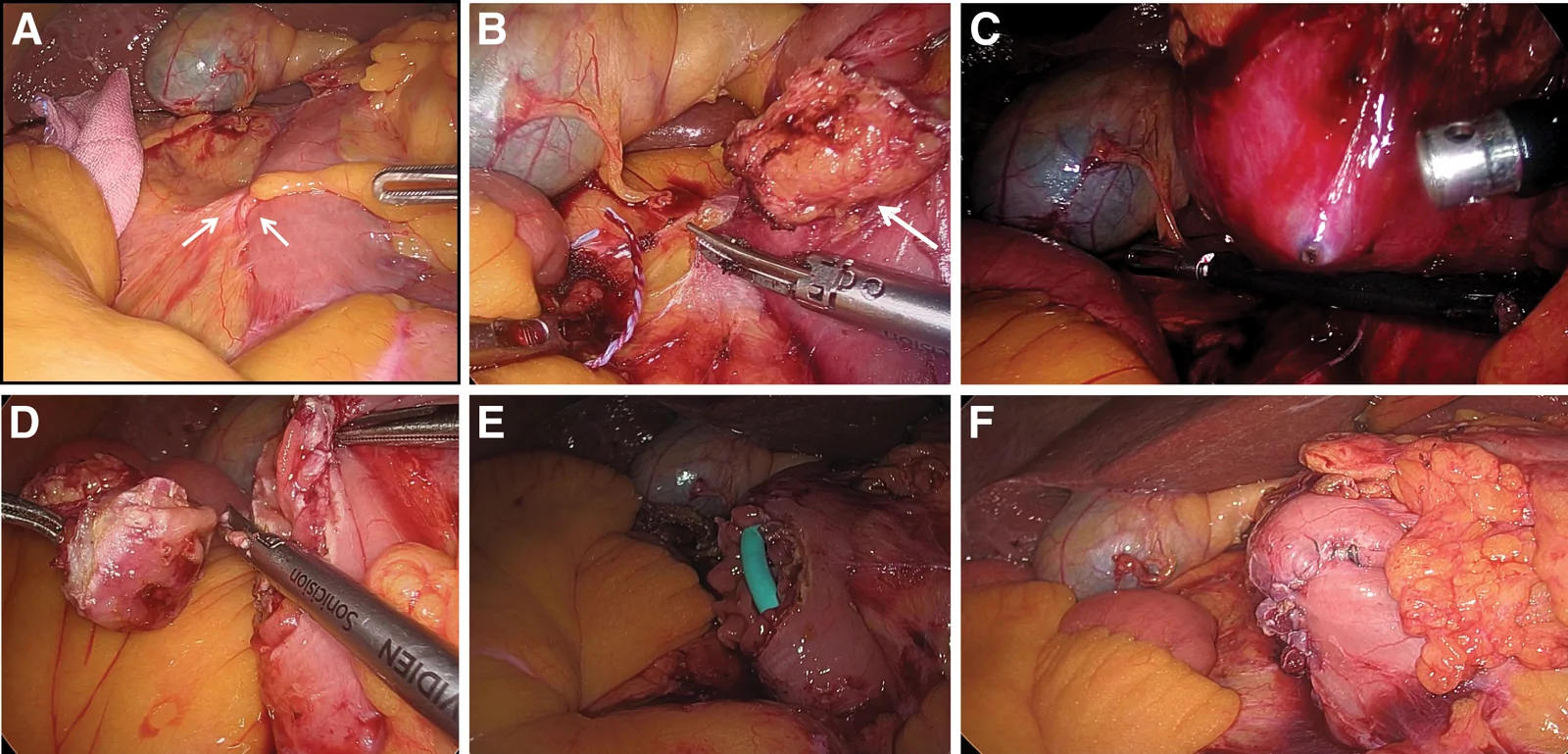
Intraoperative findings and surgical procedures. (**A**) Color changes and a firm nodule were observed at the boundary between the duodenal wall and the peritoneum (arrows). (**B**) Mobilization of the duodenum from the retroperitoneum. The recurrent tumor was covered with adipose tissue (arrow). (**C**) Endoscopic incision of the duodenal wall around the tumor. (**D**) The tumor was resected along the endoscopically determined resection line using ultrasonic coagulating shears. (**E**) A duodenal balloon catheter was inserted to maintain luminal patency and decompress the duodenum during closure. (**F**) The duodenal wall defect was closed using a laparoscopic hand-sewn suturing technique.

Endoscopic observation identified the tumor and demonstrated that its intraluminal extent was wider than anticipated from the serosal findings alone. A full-thickness incision was initiated endoscopically on the anal side of the lesion and extended toward the oral side (**Fig. 6C**). The incision lines were then connected under laparoscopic guidance using ultrasonic coagulating shears, permitting en bloc full-thickness resection (**Fig. 6D**). A duodenal balloon catheter was inserted endoscopically to maintain luminal patency during closure and reduce the risk of stenosis (**Fig. 6E**). The defect was closed in the short-axis direction by continuous full-thickness suturing using 3-0 absorbable sutures. Intraoperative endoscopy confirmed adequate luminal patency without stenosis, and the leak test showed no air leakage (**Fig. 6F**). The key operative steps are shown in **Supplementary Movie 1** (Additional File 1). Histopathological examination revealed adenocarcinoma predominantly involving the submucosa and muscularis propria, without involvement of the duodenal mucosa (**Fig. 7A**, **7B**). The resected duodenal tumor measured 13 × 11 mm. Histopathological examination revealed an intermediate infiltrative growth pattern (INFb), with no lymphatic or venous invasion (Ly0, V0). The muscularis propria adjacent to the tumor was inverted toward the duodenal lumen. The postoperative course was uneventful, and the patient has remained disease-free for more than 5 years after resection of the duodenal recurrence.

**Fig. 7:**
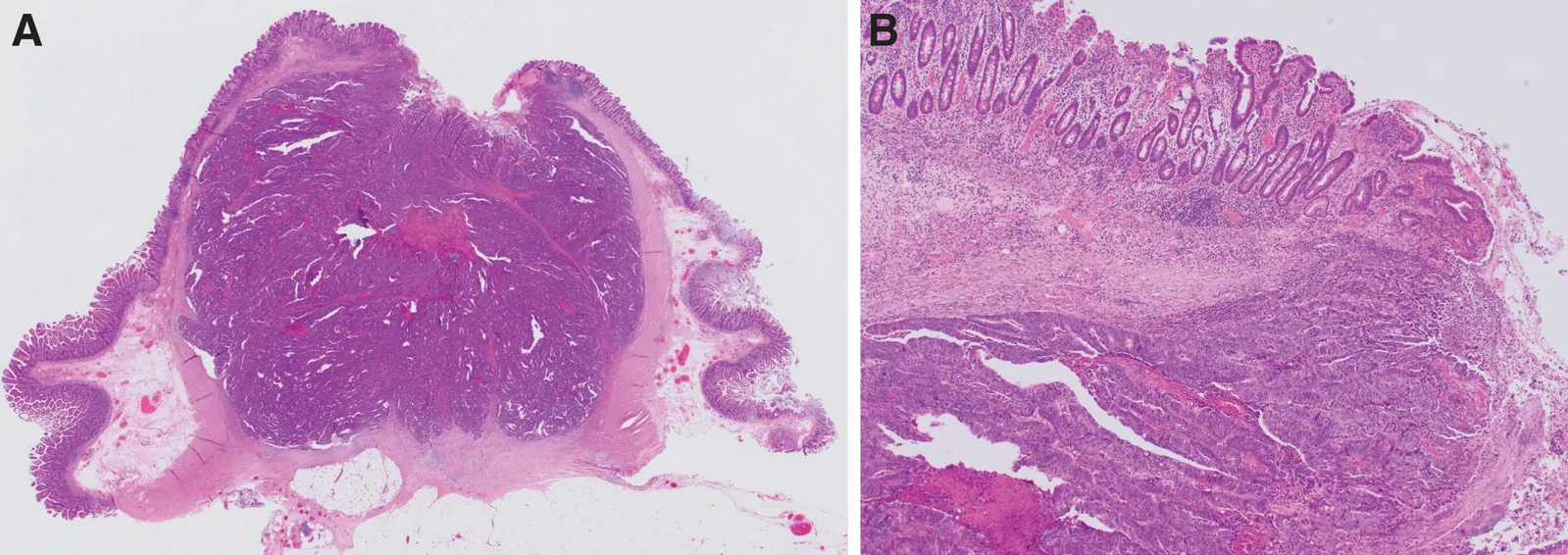
Histopathological findings of the resected specimen. (**A**) Panoramic view of the hematoxylin and eosin-stained section showing adenocarcinoma predominantly involving the submucosa and muscularis propria. The adjacent muscularis propria is inverted toward the duodenal lumen, consistent with inward progression from the outer aspect of the duodenal wall. (**B**) Higher-magnification view demonstrating fused tubular proliferation of intestinal-type adenocarcinoma similar to that of the primary colon cancer. No adenocarcinoma is observed in the duodenal mucosa (hematoxylin and eosin [H&E] staining, ×4).

## DISCUSSION

The principal clinical significance of this case is that a small, solitary postoperative recurrence involving the duodenal wall was detected during surveillance and completely removed by local full-thickness resection. Duodenal involvement by right-sided colon cancer has been described mainly in the setting of direct invasion at the primary operation.^7,8)^ In contrast, an isolated metachronous lesion appearing within the duodenal wall after colectomy is exceptionally uncommon; only rare case reports have described isolated duodenal recurrence or metastasis after colon cancer surgery.^9)^ The absence of distant metastasis, the location adjacent to the previously dissected margin, and the subsequent long-term disease-free survival support the interpretation that this lesion represented localized regrowth rather than systemic metastatic disease.

The pathological architecture provided further evidence for the route of recurrence. The carcinoma was centered in the submucosa and muscularis propria without neoplastic involvement of the duodenal mucosa, and the adjacent muscularis propria was inverted toward the lumen. This pattern is inconsistent with a conventional primary duodenal carcinoma arising from the mucosal epithelium. It instead suggests inward progression from the outer aspect of the duodenal wall. In conjunction with the microscopically positive dissected margin on the duodenal side at the initial colectomy, the findings provide a plausible anatomical explanation: microscopic residual tumor at the prior dissection plane subsequently grew into the duodenal wall and extended toward the submucosa. Positive resection margins after colon cancer surgery have been associated with substantially higher relapse rates and poorer disease-free survival, supporting the clinical relevance of microscopic residual disease in the present case.^10)^ The concordant histology of the primary and recurrent tumors and the lack of disease elsewhere also argued against a de novo duodenal cancer or hematogenous metastasis.

Determining whether complete local excision was achievable required integration of several diagnostic modalities. Contrast-enhanced CT was used to assess the relationship between the lesion and the pancreas, regional lymph nodes, and distant organs. FDG-PET supported the isolated nature of the recurrence, while upper gastrointestinal endoscopy established its precise location opposite the major papilla and allowed histological confirmation. EUS demonstrated extension to the fourth layer and contributed to evaluation of the depth of mural involvement. Because no single modality could establish both mural extent and the absence of extra-duodenal disease, the decision to pursue local resection was based on concordant findings across the available examinations rather than on EUS alone. In the present patient, these findings indicated a solitary lesion centered in the duodenal wall without radiological pancreatic or ampullary involvement. The combined assessment therefore provided a coherent basis for judging that complete local excision was technically achievable. Upper gastrointestinal endoscopy and EUS primarily characterized the lesion from the luminal side, whereas CT and FDG-PET addressed the extramural relationship and systemic distribution of disease. This distinction was important because suitability for local resection depended not only on defining the lesion itself but also on excluding findings that would have required a more extensive operation or systemic treatment. The agreement among these examinations therefore supported both the diagnosis of a localized recurrence and the technical judgment that a limited full-thickness resection could achieve complete removal.

The combined laparoscopic and endoscopic approach offered specific advantages in translating this preoperative assessment into an oncologically adequate resection. Endoscopy provided continuous visualization of the mucosal surface and revealed that the intraluminal extent of the tumor was wider than suggested by serosal inspection. Relying on the external appearance alone could therefore have resulted in an inadequate resection line. Difficulty in determining an appropriate duodenal resection line from the serosal side has also been recognized in limited duodenal resection, and endoscopically guided localization has been used to achieve minimal but adequate full-thickness resection.^11)^ The endoscopic full-thickness incision established the margin under direct intraluminal observation, while laparoscopy permitted mobilization of the duodenum, en bloc removal of the adjacent retroperitoneal tissue where required, completion of the full-thickness resection, and controlled handling of the specimen. The complementary views allowed the resection to be sufficiently wide to secure negative margins without unnecessarily sacrificing uninvolved duodenal wall. Previous reports of limited duodenal resection and LECS similarly emphasize the importance of precise localization and margin control when organ-preserving resection is attempted.^4–6,11,12)^ This capacity to reconcile oncological clearance with preservation of duodenal anatomy was the main procedural contribution of LECS in this case.

Secure reconstruction is as important as accurate excision in duodenal local resection. The duodenal wall defect was closed in the short-axis direction to minimize luminal narrowing, using continuous full-thickness suturing with absorbable sutures. An endoscopically placed balloon catheter maintained the lumen during suturing and provided a visual and tactile guide to the appropriate degree of closure. After reconstruction, intraoperative endoscopy immediately confirmed luminal patency and excluded stenosis; insufflation also permitted direct leak testing. These technical measures are consistent with the broader safety advantages of LECS reported for duodenal tumors.

Previous reports of LECS for duodenal tumors have described the feasibility of combining endoscopic localization or resection with laparoscopic full-thickness closure.^13–15)^ Although these studies mainly concerned primary superficial duodenal tumors, they provide technical context for the present procedure. In this patient, LECS was selected not to demonstrate superiority over endoscopic resection, but because the recurrent lesion was centered within the duodenal wall and required an oncologically adequate full-thickness margin. The combined approach allowed the resection line to be defined from the luminal side, the lesion to be removed en bloc under laparoscopic control, and the reconstructed lumen to be assessed endoscopically before completion of the operation. The pathological finding that the recurrent tumor involved the submucosa and muscularis propria further supports the appropriateness of a full-thickness rather than purely mucosal resection in retrospect. At the time of surgery, however, the operative plan was based on the preoperative evidence of a localized mural lesion and the expectation that an adequate margin could be obtained without pancreaticoduodenectomy. Thus, the procedure was selected to match the anticipated depth and location of disease while preserving uninvolved duodenum.

The postoperative setting introduced technical difficulties that are not encountered to the same extent in primary superficial duodenal tumors. Dense adhesions between the duodenum and adjacent jejunum restricted mobilization and obscured the external boundary of the lesion. Careful dissection was required, and a portion of the retroperitoneal tissue was intentionally retained on the duodenal wall and removed en bloc to avoid exposing the suspected recurrence. Published series of limited duodenal resections have emphasized the need for an adequate full-thickness margin and reliable primary closure.^11,12)^ In postoperative cases, adhesions may obscure the external dissection plane even when the intraluminal tumor margin is well defined endoscopically. The laparoscopic dissection should therefore be adjusted to the actual tissue planes while preserving the endoscopically determined resection margin. Close coordination between the endoscopist and laparoscopic surgeon is essential to achieve an adequate resection margin under these conditions.

The present experience does not imply that every duodenal recurrence is suitable for this approach. Local full-thickness resection should be considered only when the lesion is solitary, complete excision with negative margins is technically attainable, and preoperative imaging shows no disseminated disease or involvement that would preclude safe local resection. Particular caution is required for lesions close to the major papilla, those with extensive circumferential involvement, and those in which the relationship to the pancreatic parenchyma cannot be clearly defined. Prospective studies of LECS for primary duodenal tumors have evaluated carefully selected lesions using predefined treatment protocols, supporting the importance of rigorous selection when this technique is applied.^16,17)^ These selection principles are also relevant when applying laparoscopic and endoscopic cooperation to a recurrent lesion, although the oncological context is different.

More than 5 years of disease-free survival after resection is an important feature of this case. A single case cannot establish the oncological efficacy of LECS for colorectal cancer recurrence, and the favorable outcome may reflect the unusual biology of a localized recurrence arising from microscopic residual disease. Nevertheless, the course demonstrates that durable control is possible when rigorous surveillance detects recurrence before dissemination and complete resection is achieved. A recent report of an isolated duodenal metastasis from colon cancer also described R0 partial duodenectomy followed by recurrence-free survival, further suggesting that complete surgical removal can provide meaningful disease control in highly selected patients with isolated duodenal involvement.^9)^ The report also has inherent limitations: the indication was determined individually, the pathological mechanism of recurrence cannot be proven with absolute certainty, and evidence from primary duodenal tumors cannot be directly extrapolated to postoperative colorectal cancer recurrence. Further accumulation of similarly documented cases is required to clarify the oncological role of local resection for this rare pattern of recurrence.

## CONCLUSIONS

This case demonstrates that laparoscopic and endoscopic cooperation can facilitate complete full-thickness local resection of an isolated duodenal recurrence after colon cancer surgery. Precise preoperative staging, endoscopic delineation of the intraluminal margin, laparoscopic control of the external dissection and closure, and confirmation of luminal patency were essential to achieving complete resection while preserving duodenal continuity. Long-term disease-free survival suggests that local treatment may provide durable control when recurrence is detected before dissemination and R0 resection is technically achievable.

## SUPPLEMENTARY MATERIALS

Supplementary Movie 1 Laparoscopic and endoscopic cooperative surgery for localized duodenal recurrence. The video demonstrates the key operative steps. After careful adhesiolysis and mobilization of the duodenum, intraoperative endoscopy identified the lesion and delineated the resection line. A full-thickness incision was initiated endoscopically from the anal side and extended toward the oral side. The incision lines were connected laparoscopically using ultrasonic coagulating shears, enabling en bloc full-thickness resection. A duodenal balloon catheter was inserted to maintain luminal patency, and the defect was closed in the short-axis direction by continuous full-thickness suturing using 3-0 absorbable sutures. Intraoperative endoscopy confirmed adequate luminal patency without stenosis, and the leak test was negative.

## DECLARATIONS

### Availability of data and materials

Not applicable because no datasets were generated or analyzed during the current study.
